# Outcome of the first 200 patients with prostate cancer treated with MRI-Linac at Assuta MC

**DOI:** 10.3389/fonc.2023.1151256

**Published:** 2023-03-23

**Authors:** Or Gelbart Pridan, Merav Akiva Ben David, Svetlana Zalmanov, Yoav Lipski, Vladislav Grinberg, Daphne Levin, Sara Apter, Michal Guindi, Dan Epstein, Roman Radus, Orit Arsenault, Keren Hod, Qusai Tamami, Raphael Pfeffer

**Affiliations:** ^1^ The Adelson School of Medicine, Ariel University, Ariel, Israel; ^2^ Radiation Oncology Department, Assuta Medical Center, Tel Aviv, Israel; ^3^ Faculty of Health Sciences, Ben-Gurion University of the Negev, Beer Sheva, Israel; ^4^ Sackler School of Medicine, Tel Aviv University, Tel Aviv, Israel; ^5^ Innovation Division, Assuta Medical Center, Tel Aviv, Israel; ^6^ Radiation Oncology Department, Rabin Medical Center, Petah-Tikva, Israel; ^7^ Department of Academy and Research, Assuta Medical Center, Tel Aviv, Israel

**Keywords:** prostate cancer, MRI-Linac, SBRT, radiation therapy, MRgRT

## Abstract

**Background:**

We present our experience with MR-guided stereotactic body radiotherapy (SBRT) for 200 consecutive patients with prostate cancer with minimum 3-month follow-up.

**Methods:**

Treatment planning included fusion of the 0.35-Tesla planning MRI with multiparametric MRI and PET-PSMA for Group Grade (GG) 2 or higher and contour review with an expert MRI radiologist. No fiducials or rectal spacers were used. Prescription dose was 36.25 Gy in 5 fractions over 2 weeks to the entire prostate with 3-mm margins. Daily plan was adapted if tumor and organs at risk (OAR) doses differed significantly from the original plan. The prostate was monitored during treatment that was automatically interrupted if the target moved out of the PTV range.

**Results:**

Mean age was 72 years. Clinical stage was T1c, 85.5%; T2, 13%; and T3, 1.5%. In addition, 20% were GG1, 50% were GG2, 14.5% were GG3, 13% were GG4, and one patient was GG5. PSA ranged from 1 to 77 (median, 6.2). Median prostate volume was 57cc, and 888/1000 (88%) fractions required plan adaptation. The most common acute GU toxicity was Grade I, 31%; dysuria and acute gastrointestinal toxicity were rare. Three patients required temporary catheterization. Prostate size of over 100cc was associated with acute fatigue, urinary hesitance, and catheter insertion. Prostate Specific Antigen (PSA) decreased in 99% of patients, and one patient had regional recurrence.

**Conclusion:**

MR-guided prostate SBRT shows low acute toxicity and excellent short-term outcomes. Real-time MRI ensures accurate positioning and SBRT delivery.

## 1 Introduction

Prostate cancer (PC) is the second most frequent cancer in men worldwide, usually in men age 50 and older. Every year, 1,400,000 new patients are diagnosed with PC and 375,000 patients die (1). The majority (91%) of PCs are diagnosed at a local or regional stage, for which the 5-year survival rate approaches 100%. The 5-year survival for disease diagnosed at metastatic stage is 30% and the 10-year survival rate for all stages combined is 98% (2). Early PC treatment options include active follow-up (active supervision/surveillance), surgery (prostate gland removal), and radiation therapy (RT; external beam radiation or brachytherapy) (3). RT is a key modality in the treatment of patients with low-, intermediate-, and high-risk PC. This non-invasive technique can be offered to many patients with PC with minimal side effects in the modern radiation era (4).

The most commonly available RT technique is external beam RT (5). The fundamental problem in treating localized PC is to provide a curative dose of radiation to the prostate while reducing the exposure to healthy surrounding organs such as the bladder, rectum, and femoral heads (5). This issue is largely overcome with modern radiation techniques, which include three-dimensional (3D) conformal radiotherapy, intensity-modulated RT, and volumetric modulated arc therapy combined with image guidance have allowed delivery of larger radiation doses with lower toxicity (6). Androgen deprivation therapy (ADT) is usually kept for clinically localized, unfavorable intermediate to high-risk PC (3)

Since August 2019, a gantry-based MR-guided linear accelerator (MRgRT) is in use at our institution (ViewRay MRIdian). This device integrates full 3D MRI target identification and radiation dose replanning before every treatment (7, 8). In our study, we collected the clinical and treatment data for the first 200 patients with PC treated with MRgRT in our institution, as well as their outcome measures. This will be one of the first reports and the largest series as of today regarding PC treated on an MR-Linac.

## 2 Materials and methods

This descriptive study reports 200 consecutive patients with localized PC who were treated with MRI-Linac at our institution between August 2019 and July 2021. The study was approved by the local Institutional Review Board (IRB). Inclusion criteria were histologically proven PC, localized PC by imaging, and at least 3-month follow-up. Patients treated for local recurrence following former prostatectomy or prior radiation or who had received previous pelvic RT for any cause were excluded from this analysis.

Patients underwent MRI simulation on the MRI linear accelerator (supine position, both arms on the chest, two glycerin suppositories 4 h prior to simulation and 400 cc water PO 45 min prior to scanning and before each fraction) followed by CT-based simulation in the same position. The treatment planning included fusion with pre-treatment imaging [MRI and/or positron emission tomography - prostate-specific membrane antigen (PET-PSMA)] and contouring target volume [prostate contouring target volume (CTV)] and OARs—rectum, bladder, and femoral heads, on the MR simulation imaging. The urethra was not contoured. All contours underwent an expert radiologist review prior to planning (SA). Prescription dose was 36.25 Gy in 5 fractions delivered over 2 weeks (alternate days) to the entire prostate with 3-mm margins (PTV); no regional nodes were treated. Doses to target volume and OAR were evaluated using institutional constrains as shown in **Table 1**. One patient received GTV boost to 40 Gy. On each fraction, MRI was performed, the OARs and prostate were re-contoured accordingly, and plan was adapted if tumor and OAR doses were significantly worse than the simulation-based plan or did not match the constrains see **Figure 2**. During radiation, the prostate was monitored with real-time (four frames/s) single-frame MRI, and treatment was automatically interrupted if the target volume moved out of the PTV range by 5%.

**Table 1:** Target volume and organs at-risk constrains for treatment plans.

Structure	Dosimetric index (volume)	Accepted criteria (Gy)
PTV	≥95%	34.4
Rectum	Max point dose	38.0
	<1.0 cc	36.25
	<3.0 cc	34.43
	<10 cc	32.62
Bladder	Max point dose	39.4
	<0.1 cc	38.0
	<1.0 cc	36.25
	<15.0 cc	32.62
Femoral heads	<10 cc	30.0

Treating physicians monitored patients’ side effects both throughout and after the course of treatment. Gastrointestinal (GI) (diarrhea, proctitis, tenesmus, rectum numbness, hemorrhoids, encopresis, incontinence, pain), urinal (dysuria, increased frequency of urination, nocturia, urinary jet strength, urgency, and nonspecific urination complains) and general side effects (fatigue) were monitored during radiation and physician reported in every follow-up visit thereafter. Side effects were rated according to CTCAE (version 5) (9). In addition, patients’ PSA levels were tracked at baseline and every 3–6 months.

### 2.1 Data analysis

The primary endpoint was to evaluate GI and urinary side effects. Secondary endpoint was a short-term treatment outcome. Associations between patients’ characteristics, treatment characteristics, and side effects were evaluated by Mann–Whitney test, Spearman correlation, and Chi-square test, as appropriate. A linear mixed model for repeated measure analysis was used to evaluate individual PSA levels throughout the study follow-up within the non-ADT population. In addition, four linear mixed models with adjustments for potential confounders were used. The first model adjusts for patients’ age and interaction with time (i.e., age × time); the second model adjusts for prostate size and interaction with time (i.e., prostate size × time); the third model adjusts for International Society of Urological Pathology (ISUP) and interaction with time (i.e., ISUP × time); and the fourth model adjusts for ISUP, interaction with time (i.e., ISUP × time), prostate size, and interaction with time (i.e., prostate size × time). To avoid multicollinearity, we verified that there are no correlations between these independent variables that were included in each of the models; therefore, in the last model, age and its interaction with time were not included with all the rest of the independent variables (i.e., prostate size, prostate size × time, ISUP, and ISUP × time). All of these adjustments were defined as fixed effects in all models.

Level of significance used for all analyses was two tailed and set at *p* < 0.05. The SPSS statistical package (version 28, SSPS Inc., Chicago, IL) was used for all statistical analyses.

## 3 Results

### 3.1 Patients and treatment characteristics

With median follow-up of 16.4 months (range, 3–35.9 months), our first 200 consecutive patients were evaluated. **Table 2** summarizes patient and treatment characteristics. Clinical stage was I, 85.5% (n = 171); II, 13.0% (n = 26); and III, 1.5% (n = 3). The average percentage of positive biopsy cores was 40.8%, and 64 (32.0%) of the patients had involvement of ≥50.0% cores. ISUP 1 accounted for 20.5% (n = 41) of patients, 50.0% (n = 100) had ISUP 2, 14.5% (n = 29) ISUP 3, 13.0% (n = 26) ISUP 4, and only 0.5% (n = 1) with ISUP 5 (three patients had missing data). Over 25% had prostate volume of 67.8cc and 23 (11.5%) had prostate larger than 80cc. Prostate size was not associated with ISUP. In this cohort, 92.0% (N = 184) had a diagnostic multiparametric MRI (1.5 or 3 Tesla) prior to radiation, and the majority (91.6%) had PIRADS 4/5 lesions, 6% had PIRADS 3, 1.8% PIRADS 2, and 0.6% PIRADS 1. MRI showed suspected extracapsular extension in 19.5% (n = 39), neuro-vascular bundle (NVB) involvement in 9.5% (n = 19), and seminal vesicles involvement in 4.3%. PET-PSMA was available for 79.0% (n = 158) of the patients before RT.

**Table 2:** Patients’ characteristics.

Age (years), median (range)	72.0 (53.0-90.0)
BMI (kg/m^2^), median (range)	26.9 (19.8-37.7)
Prostate size (cc), median (range)	53.3 (16.5-171.8)
PSA at diagnosis (ng/ml)*,**, median (range)	6.3 (1.4-72.0)
Treatment period (days), median (range)	10.0 (7.0-29.0)
Androgen deprivation therapy, n (%)	56 (28.0)

BMI, body mass index; PSA, prostate specific antigen.

*Available for 180 patients up to 4 months prior to RT.

**PSA at diagnosis includes ADT group and non-ADT group.

Mean treatment time (from closing doors to end of treatment: re-sim, contour check, plan evaluation, optimization and re-calculation when appropriate, on-board QA, actual treatment time) was approximately 50 min. In addition, 888/1000 (88.8%) daily treatments required plan adaptation. No correlation was found between adaptation required and time interval between simulation and RT. There was no association between age, PSA level at diagnosis, prostate volume, and body mass index (BMI) to number of adaptations.

### 3.2 Toxicity

We analyzed two groups of side effects: acute side effects (during and up to 30 days following the end of radiation) and subacute side effect (30–90 days following radiation). During radiation period, 0.5% (n = 1) reported fatigue, whereas 9.0% (n = 18) reported on fatigue later in the follow-up visit. The most common genitourinary (GU) symptom reported was mild dysuria (grade I) by 31.0% (n = 62) of patients, subsiding to 11% (n = 22) by 3 months. In addition, 20.5% (n = 41) reported increased nocturia (grade I) and increased frequency (grade I), subsiding to 13.0% (n = 26) and 3.0% (n = 6), respectively, by 3-month follow-up. In addition, 2.5% (n = 5) reported urgency grade I in the subacute period. Three patients (1.5%) needed a catheter insertion (grade II) (9) during radiation treatment: in one patient after 1 fraction and in two patients following 3 fractions. All catheters were removed successfully 1, 7, and 10 days following RT. Prostate mean volume for patients with catheter was significantly higher, 105 cc (85, 89, and 141 cc) vs. 65.7 cc (*p* = 0.029). Catheter insertion was not associated with age, PSA level at diagnosis, or BMI.

Patients older than 75 years reported higher incidence of acute nocturia (*p* = 0.004). Larger prostate size was associated with acute fatigue 107.6 cc vs. 53.7 cc (*p* = 0.037) and with acute/subacute sensation of urinary hesitance of 89.0 and 109.4cc, vs. 53.3 and 52.6 cc, respectively (*p* = 0.0006 and *p* = 0.014). In addition, BMI was significantly higher among those who reported subacute penile pain [34.5 kg/m (2)], compared to those who did not (26.9 kg/m (2); *p* = 0.005).

The reported GI side effects were minor (**Table 3**). Maximal grade was II in this cohort. No patient reported constipation, encopresis, nausea, or any other abdominal/GI-associated symptoms.

**Table 3:** Acute and subacute gastrointestinal toxicity.

Side-effect	Acute	Sub-acute
Hemorrhoids, n (%)	5 (2.5)	0
Anal pain, n (%)	5 (2.5)	2 (1.0)
Tenesmus, n (%)	5 (2.5)	1 (0.5)
Incontinence, n (%)	0	0
Proctitis, n (%)	2 (1.0)	0 (0.0)
Diarrhea, n (%)	1 (0.5)	0

ADT, received by 28.6% of our cohort, was not associated with acute or subacute GU or GI side effects.

### 3.3 Outcome

Pre-treatment PSA ranged from 1.4 to 72.0 ng/ml (**Table 2**) and was ≥10 ng/ml in 34 patients (17.0%). Only 28.6% (N = 57) received ADT in this cohort.

**Figure 1:**
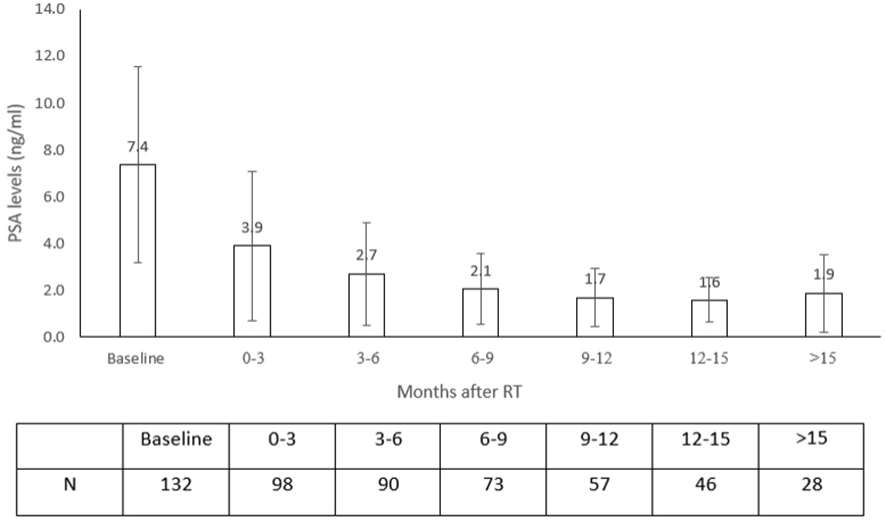
PSA during the study follow-up in non-ADT patients.

In the non-ADT group (71.4%, N = 142), mean baseline PSA was 7.4 ng/ml and decreased with time (*p*<0.001) (**Figure 1**). PSA decline rate was 1.0 ng/ml per 3 months on average. PSA reduction was not associated with either age, prostate size, or ISUP.

**Figure 2:**
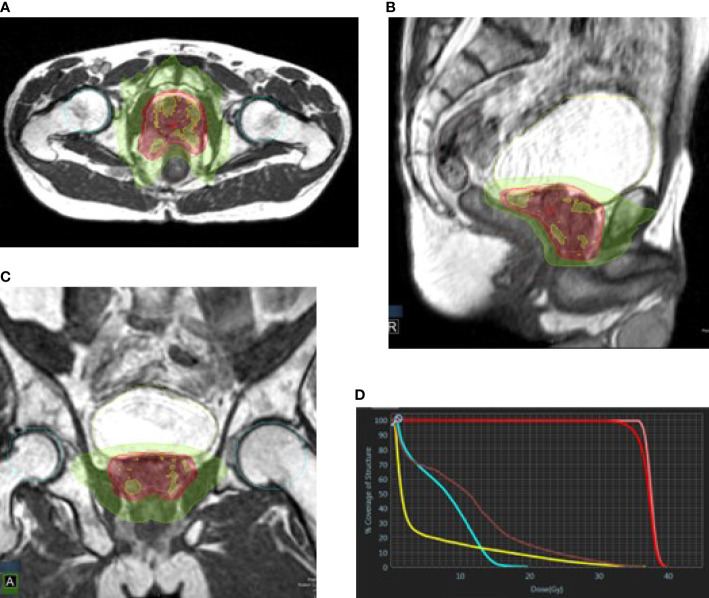
Prostate plan in axial **(A)**, sagittal **(B)**, and coronal **(C)** views. Green, 50% isodose line; red, 95% isodose line; yellow, 105% isodose line. **(D)** DVH: pink, CTV; red, PTV; yellow, bladder; brown, rectum; cyan, femoral heads.

The PSA nadir in our non-ADT cohort has not been reached due to the short follow-up period; however, 6% were below 0.2 ng/ml and 41% had PSA level of <1 ng/ml at the last follow-up.

Of the 200 patients treated, 184 (92.0%) were available for follow-up of at least 6 months (3–35.9 months) following RT. One patient died because of cardiac arrest, and one (0.5%) had an isolated regional recurrence (isolated ileac lymph node) 7 months following MRgRT, treated with comprehensive pelvic irradiation, and is free of disease at the time of this analysis. A total of 192 patients (96.0%) were NED (no evidence of disease) when performing the analysis. There were missing data for seven (3.5%) patients.

## 4 Discussion

To the best of our knowledge, this study describes the largest series of patients with PC treated with MRgRT, reporting side effects and short-term outcomes. MRgRT with daily online plan adaption is a novel strategy for administering stereotactic body radiotherapy (SBRT) for PC but requires longer treatment time and multi-professional personnel efforts (10).

In this consecutive cohort, we found low rates of GI toxicity, and, although 31% experienced GU side effects, they were transient and mostly grade I by nature. A short-term follow-up demonstrated excellent local control and reduction in the PSA level. By using adaptive planning, real-time tracking, and particularly the use of only 3-mm CTV to PTV margins, the dose to the rectum and bladder is lower, leading to these results as described by others as well (11–13). Most CT-based prostate SBRT series use larger margins (14–16). The Magnetic resonance imaging-guided stereotactic body radiotherapy for prostate cancer (MIRAGE) study used 2-mm margins for MR-guided treatment and 4-mm margins for CT-guided treatment (13).

### 4.1 Acute side effects

In our series, 31% and 6.5% experienced grade I (mostly mild dysuria) or grade II GU toxicity, respectively, and three patients needed catheter insertion. For GI toxicity, very few patients experienced any side effects, with mostly grade I reported. Our excellent toxicity profile is lower when compared with other studies reporting acute GU and GI toxicity with MRgRT (11, 12).

In the early pioneer reported series of MRgRT, Alongi et al. and Tetar et al. reported very low GI and GU toxicity in their series, demonstrating the feasibility and safety of this extreme hypofractionated RT protocol (17, 18). In a study by Bruyzeel (11) et al., their group described meticulous patient-reported outcome measure and clinician reported outcome measure outcomes of 104 patients with the same radiation protocol of 36.25 Gy in 5 fractions using MRgRT, reporting ≥ grade II of any acute GU side effects of 23.8% and 5% GI. Ugurluer et al. in their series of 50 patients with a similar RT protocol reported 28% of grade I GU toxicity and 36% of grade II (12). Only 6% experienced grade I GI toxicity. The low rates of GI toxicity and the moderately low rates of GU toxicity are consistent in all studies. All these groups used 3 mm around the GTV for PTV delineation and same OAR constrains with daily adaptation. The MIRAGE study, a randomized phase 3, compared MRgRT vs. CT-based SBRT in PC with 40 Gy in 5 fractions (13). Two-millimeter margins were used for the non-adaptive MRgRT and 4-mm margins for CT-based treatment. In their study, they report significantly lower incidence of acute GI and GU side effects in the group treated with MRgRT (13).

In series using similar but non-MRgRT SBRT protocols of 36.25–40 Gy, higher acute side effects were reported. In the series of 309 patients with real-time tracking of implanted fiducials by Meier et al., 59% experienced grade I GU toxicity and 26% grade II, with 55% and 8.1% GI toxicity, respectively (19). These results resemble the findings in the study reported by Brand et al. In their study of 874 patients, half received conventional fractionated/moderately hypofractionated RT compared to SBRT (14). In 415 patients in the SBRT arm (36.25 Gy, 5 fractions), they reported that 57% of the patients experienced grade I GU toxicity and 21% grade II with 2% grade III and two patients with grade IV (14). For GI toxicity, 53% grade I and 10% grade II with one patient experience grade III.

Three patients needed catheter insertion during radiation in our series (1.5%), reported as grade II by CTACE Vr. 5. In our cohort, the urethra was not delineated and was not accounted for during dose calculation. We identified high prostate volume as risk factor for urinary retention in these patients. In the 104 patients, Bruynzeel et al. reported that the treatment was delivered to the prostate with simultaneous integrated relative sparing of the urethra, and no patient needed a catheter insertion (11). One patient (2%) in the study by Ugurluer et al. needed a catheter during RT (12). In a non-MRgRT prostate SBRT series, the need for catheterization was 1% in the acute phase period (19). Urethral sparing techniques may reduce urinary symptoms as reported from brachytherapy series (15, 20); however, daily urethral catheter insertions were uncomfortable and may increase urinary tract discomfort. In addition, urethral sparing based on catheter-free MRI can be observer-dependent, time-consuming, risk-underdosing tumor close to the urethra and produce heterogeneity in the target volume (16). There are no reliable urethral contouring guidelines, and it is not consistently delineated in most series (19). Of note, the low rates of GI toxicity are comparable to the reports of external beam RT using rectal spacers, showing less than 5% grade II GI toxicity (21). Another advantage of the MRgRT is the avoidance of the need for implanted fiducials, i.e., gold markers.

Overall, the incidence of side effects was low, and the treatment was well tolerated in prior reports of prostate SBRT treated by MRgRT, leading to worldwide acceptance of extreme hypofractionated protocols for localized PC, following the road for shorter radiation protocols as HYPRO and CHHip trials (11–13, 22, 23).

With median follow-up of 16.4 months, only one patient (0.5%) experienced regional recurrence of isolated ileac lymph node and was salvaged by comprehensive pelvic irradiation. This low rate of regional recurrence, although very early, is anticipated, as most of the patients were in the low-risk group (ISUP 1 + 2, 70.5%). PSA level as a surrogate for distant metastatic disease and overall survival is well established in definitive radiation to the prostate gland (24, 25). There is a range of possible PSA level measurements in the literature as a cut-point value for nadir determination, and as reported, 41% of our cohort reached a PSA level lower than 1 ng/ml (24). Because the nadir of PSA level was not yet achieved in our series, with our reported mean decrease rate of 1 ng/ml every 3 months, a longer follow-up is necessary for evaluating this outcome.

We acknowledge the limitation of this study as this cohort is a single institution series, retrospective in nature, with a relatively short follow-up time. However, this is the largest study evaluating short-term toxicity and outcome of patients with localized PC treated with MRgRT with an ultra-hypofractionated scheme. In this homogenous group, disease baseline imaging data were very updated with 92% undergoing diagnostic prostate MRI prior to RT and PET-PSMA available for 79% of the patients. A total of 97% of the patient were available for follow-up.

## 5 Conclusions

MRgRT for localized PC with ultra-hypofractionated dose protocol of 36.25 Gy demonstrated low rates of acute and subacute GI and GU toxicity with excellent short-term outcomes. We anticipate that future research will add to our understanding of the tolerability and clinical outcomes of this novel technology method.

## Data availability statement

The raw data supporting the conclusions of this article will be made available by the authors, without undue reservation.

## Ethics statement

The studies involving human participants were reviewed and approved by the Institutional Review Board (or Ethics Committee) of ASSUTA Ramat Hahayal (ASMC-0062-21 19.12.2021). Written informed consent for participation was not required for this study in accordance with the national legislation and the institutional requirements.

## Author contributions

The authors confirm the contribution to the paper as follows: OG: data collection, data analysis and interpretation of results, and draft manuscript preparation; MB: treating physician, data analysis and interpretation of results, draft manuscript preparation, design of the work, and supervision; SZ: treating physician, and writing review and editing; YL: treating physician, and writing review and editing; VG: treating physician, and writing review and editing; DL: on-board physicist and analyst, and data collection; SA: delineation quality assurance and data analysis; MG: data analysis; DE: on-board physicist and analyst, and data collection; RR: on-board physicist and analyst, and data collection; OA: on-board physicist and analyst, and data collection; QT: on-board physicist and analyst, and data collection; KH: statistician, data management, data analysis, and interpretation of results; RP: treating physicians, critical revision of the article, and supervision. All authors have read and agreed to the published version of the manuscript.
